# Supplementary material for: APOBEC3B Gene Expression in Ductal Carcinoma In Situ and Synchronous Invasive Breast Cancer
Source: Cancers (Basel). 2019 Jul 27;11(8):1062. doi: 10.3390/cancers11081062 (PMC6721358; doi:10.3390/cancers11081062)
Supplement: Supplementary file 1 [file cancers-11-01062-s001.pdf]

Supplementary Materials: *APOBEC3B* Gene Expression in Ductal Carcinoma In Situ and Synchronous Invasive Breast Cancer

Table S1. Performance of the Taqman mRNA assays used in this study.

mRNA Assay	Start CDNA			Start RNA		
	Slope	% Efficiency	R^2	Slope	% Efficiency	R^2
GUSB	−3.34	99.6%	0.94	−3.63	94.3%	0.92
HMBS	−3.53	96.0%	0.96	−3.10	96.0%	0.87
Mean GUSB+HMBS	−3.44	97.6%	0.96	−3.37	99.0%	0.90
APOBEC3B	−3.28	100.9%	0.92	−3.19	102.9%	0.88
EPCAM	−3.24	101.8%	0.94	−3.06	106.1%	0.98
PTPRC	−3.21	102.4%	0.91	ND	ND	ND
	7 individual experiments with 5 dilutions (0.4 - 100 ng/µL input)			*7 individual experiments with 3 dilutions (0.62-10 ng/µL)*		
